# Preparation and Quality Control of the [^153^Sm]-Samarium Maltolate Complex as a Lanthanide Mobilization Product in Rats

**DOI:** 10.3797/scipharm.1011-08

**Published:** 2011-02-24

**Authors:** Zohreh Naseri, Amir Hakimi, Amir R. Jalilian, Ali Nemati Kharat, Ali Bahrami-Samani, Mohammad Ghannadi-Maragheh

**Affiliations:** 1 Inorganic Chemistry Department, Faculty of Sciences, Tehran University, Tehran, Iran; 2 Radiopharmaceutical Research and Development Lab (RRDL), Nuclear Science and Technology Research Institute (NSTRI), Tehran, Iran

**Keywords:** Detoxification, Maltolate, Sm-153, Biodistribution, Radiolabeling

## Abstract

Development of lanthanide detoxification agents and protocols is of great importance in management of overdoses. Due to safety of maltol as a detoxifying agent in metal overloads, it can be used as a lanthanide detoxifying agent. In order to demonstrate the biodistribution of final complex, [^153^Sm]-samarium maltolate was prepared using Sm-153 chloride (radiochemical purity >99.9%; ITLC and specific activity). The stability of the labeled compound was determined in the final solution up to 24h as well as the partition coefficient. Biodistribution studies of Sm-153 chloride, [^153^Sm]-samarium maltolate were carried out in wild-type rats comparing the critical organ uptakes. Comparative study for Sm^3+^ cation and the labeled compound was conducted up to 48 h, demonstrating a more rapid wash out for the labeled compound. The effective and biological half lives of 2.3 h and 2.46h were calculated for the complex. The data suggest the detoxification property of maltol formulation for lanthanide overdoses.

## Introduction

Lanthanides have been used extensively in industry, ophthalmic and camera lenses, petroleum cracking, nuclear reactors, television tubes, and mirrors, to mention a few applications. Symptoms of acute samarium (and other lanthanides) intoxication include defecation, writhing, ataxia, sedation, and labored respiration, which can happen in accidents in reactors and other related industries.

However, not much data are available on the lanthanide detoxification processes of the subjects after acute exposures. Developing lanthanide mobilization protocols using nontoxic and human approved chelators is an interesting field in human health.

**Fig. 1.**
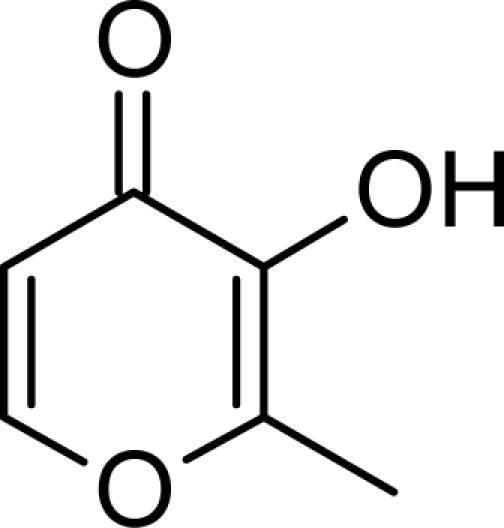
Chemical structure of maltol

Maltol (3-hydroxy-2-methyl-4-pyrone), a common product formed upon heating of carbohydrates, is an approved food additive, used to impart a desirable malty taste and odor to breads, cakes, beer and other beverages (Figure 1). Maltol loses its hydroxyl proton at neutral to basic pH levels, forming the maltolate anion; this anionic molecule forms a strong bidentate/tridentate chelate with gallium, iron, zinc, aluminum, vanadium [1] and lanthanides [2].

Some of maltolate metal complexes are reported as biologically active compounds including gallium-maltolate for lymphoma treatment [3]. On the other hand aluminum-maltolate complex has also demonstrated *in vitro* apoptotic cell death pathway in man [4] as well as anti-microbial effects [5].

Maltol and its derivatives have been used in treatment of iron overload disorders such as haemochromatosis and thalassemia major [6] and aluminium toxication [7].

Due to lanthanide maltol complexes stability reports [8] and the possible application of maltol as a detoxifying agent in lanthanide over-exposure, in this work, we were interested in biodistribution and effective half life determination of a final lanthanide-maltol complex, *i.e.* [^153^Sm]-samarium maltolate, as a final detoxification product in biological systems. Samarium was chosen as the appropriate lanthanide due to considerations, for instance, ^153^Sm (T_1/2_ = 46.7 h) is usually prepared by neutron activation of natural and/or enriched ^152^Sm_2_O_3_ [9] and is an excellent lanthanide radionuclide for biological studies due to high production yields in research reactors, medium energy beta particles and the existence of photopeak at 103 keV for detection [10].

Thus, in this work, the preparation, stability tests, partition coefficient determination and comparative biodistribution studies of [^153^Sm]-samarium maltolate as the final product of lanthanide detoxification have been reported.

## Results and discussion

### Production and quality control of ^153^Sm

The radionuclide was prepared in a research reactor according to regular methods with a range of specific activity 22–28 GBq/mg for radiolabeling use, after counting the samples on an HPGe detector for 5 hours, a negligible amount of impurities were recorded and shown to be Eu radionuclides.

The ^153^Sm radionuclide in specific activity of 28 GBq/mg was prepared for radiolabeling. The radioisotope diluted and evaporated to obtain the desired pH and volume was followed by sterile filtering. Radiochemical impurities in the ^153^Sm sample used in the radiolabeling step were checked by two solvent systems: A, a mixture of 10 mmol/L DTPA solution as mobile phase on Whantman No.2 paper (pH=3), the free samarium cation in ^153^Sm^3+^ form, was chelated with the polydentate eluting leading to the migration of the cation in ^153^Sm-DTPA form to higher R_f_ (retention factor), any other ionic species would lead to the observation of new radiopeaks, especially in origin. B, a mixture of 10% ammonium acetate:methanol (1:1) was used as another solvent system on the Whatman No. 2 paper, ^153^Sm^3+^ remains at the origin using this system while other ionic species would migrate to higher R_f_s (Table 1).

On the other hand, 10% ammonium acetate: methanol mixture was also used for the determination of radiochemical purity. In this solvent system, the fast eluting species were possibly Sm-153 cation, other than Sm^3+^ (2%) and the remaining fraction at R_f_.0 was a possible mixture of Sm^3+^ and/or colloids. The difference in values of impurity in two solvent systems is possibly due to the presence of colloidal impurity in the sample (2%) (Tables 1, 2).

### Preparation of ^153^Sm-MAL

In order to obtain the highest specific activity in the shortest possible time, a quantitative study was designed using different amounts of MAL and various time intervals for a specific amount of radioactivity while 60°C was considered a suitable temperature. A satisfactory labeling yield of 99–100% was obtained at this temperature using 20–30 mg of MAL within 2 h.

Because of relative lipophilic [^153^Sm]-samarium maltolate complex and participation of several polar functional groups in its structure, [^153^Sm]-samarium maltolate migrated to the solvent frontline in ITLC while ^153^Sm cation was retained in origin (Table 1). The labeling step took about 2 h. In all radiolabeling procedures (n=5), the labeling yield was over 99% (Table 2).

The partition coefficient for the labeled compound was calculated (logP. 1.869) demonstrating a rather lipophilic complex as it could be observed from the chromatographic behavior.

The final radiolabeled complex diluted in normal saline was then passed through a 0.22 μm (Millipore) filter for sterilization. Incubation of [^153^Sm]-samarium maltolate in freshly prepared human serum for 24 h at 37°C showed no loss of ^153^Sm from the complex.

### Biodistribution studies for ^153^Sm cation in wild-type rats

The animals were killed by CO_2_ asphyxiation at selected times after injection (2, 4, 24 and 48h). Dissection began by drawing blood from the aorta followed by removing the heart, spleen, muscle, bone, kidneys, liver, intestine, stomach, lungs and skin samples. The tissue uptakes were calculated as the percent of area under the curve of the related photo peak per gram of tissue (% ID/g) (Table 3).

The liver uptake of the cation is comparable with many other radio-lanthanides mimicking calcium cation accumulation; about %3 of the activity accumulates in the liver after 48 h. The transferin-metal complex uptake and the final liver delivery seems the possible route of accumulation.

The blood content is low at all time intervals and this shows the rapid removal of activity in the circulation. The lung, muscle and also skin do not demonstrate significant uptake which is in accordance with other cations accumulation. A %4 bone uptake is observed for the cation which remains almost constant after 96 h (data not shown). The spleen also has a significant uptake possibly related to reticuloendothelial uptake. The kidney plays an important role in ^153^Sm cation excretion especially after 24 h.

The accumulation of [^153^Sm]-samarium maltolate is demonstrated in Table 4. The spleen, liver and kidney were the major accumulation sites of the radiolabeled compound.

Regarding the blood activity content, the Sm cation content is almost intact in 48 hours which can be a result of metal-serum protein interactions, while in case of [^153^Sm]-samarium maltolate, the activity is rapidly decreased in 24 hours and reaches its minimum in 48 hours (Table 4). This is a significant detoxifying property for maltol in the presence of radiolanthanides.

**Fig. 3.**
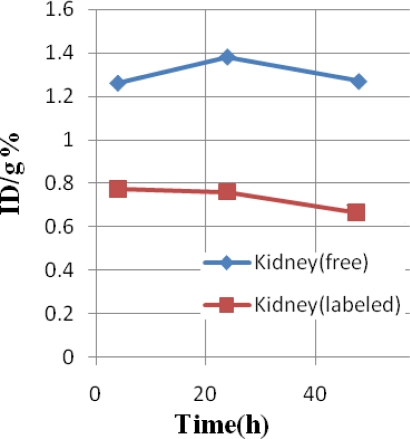
Comparative %ID/g in the kidney for [^153^Sm]-samarium maltolate (labeled, red) and ^153^SmCl_3_ (free, blue) in wild-type rats

Regarding the kidney activity content, both species are excreted from the kidneys; the excretion difference is not significant (Figure 3).

**Fig. 4.**
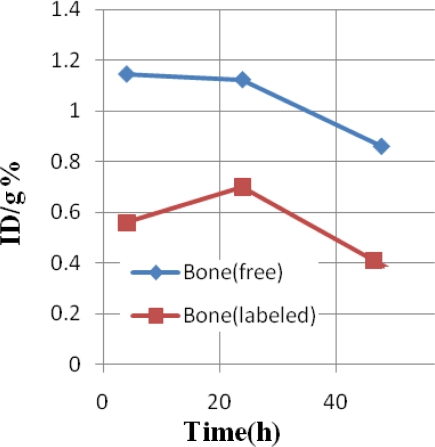
Comparative %ID/g in the thigh bone tissue for [^153^Sm]-samarium maltolate (labeled, red) and ^153^SmCl_3_ (free, blue) in wild-type rats

The bone activity content, is always a major problem in lanthanide overdose. The Sm cation content is almost higher at all time intervals compared with the labeled compound and in 48 hours almost no activity (less than 0.4%) can be observed in the bones (Fig. 4).

**Fig. 5.**
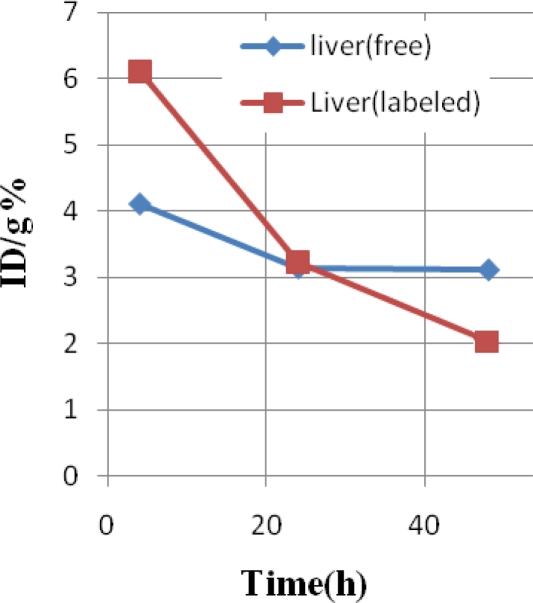
Comparative %ID/g in the liver for [^153^Sm]-samarium maltolate (labeled, red) and ^153^SmCl_3_ (free, blue) in wild-type rats

The liver activity content is another major problem in lanthanide overdoses. The Sm cation content is almost less in the first few hours while after 24 h it is constant at around %3. The whole liver activity decreases to less than %2 in 48 hours which is a satisfactory activity reduction for detoxification process (Figure 5).

Considering the whole-body activity content, an accumulative activity was calculated for the [^153^Sm]-samarium maltolate among the tissues in 4–48 time period. A polynominal curve can be fitted to the time-activity data with high R-square value (R^2^=1). Considering the half activity of the starting time interval (4h, 11.1178 %ID/g), a biological half-life for the complex can be determined by dissolving the equation (at ×=5.55). Thus the effective half life of about 2.3 h is calculated for the complex. While the biological half life can be calculated (T1/2 biol. 2.46).

## Experimental

Production of ^153^Sm was set up at Tehran Research Reactor (TRR) using ^152^Sm (n, γ) ^153^Sm reaction with ^152^Sm in purity of 98.7% (ISOTEC Inc.). Maltol was purchased from Aldrich Co., Germany, without further purification. Chromatography paper (Whatman No. 2) was obtained from Whatman (Maidstone, UK). Radio-chromatography was performed using a bioscan AR-2000 radio TLC scanner instrument (Bioscan, Paris, France). A high purity germanium (HPGe) detector coupled with a Canberra™ (model GC1020-7500SL) multichannel analyzer and a dose calibrator ISOMED 1010 (Dresden, Germany) were used for counting distributed activity in rat organs. All other chemical reagents were purchased from Merck (Darmstadt, Germany). Calculations were based on the 103 keV peak for ^153^Sm. All values were expressed as mean ± standard deviation (Mean ± SD) and the data were compared using Student’s T-test. Statistical significance was defined as P<0.05. Animal studies were carried out in accordance with the United Kingdom Biological Council's Guidelines on the Use of Living Animals in Scientific Investigations, 2nd ed. Male healthy rats were purchased from Pasteur Institute, Tehran, Iran.

### Production and quality control of ^153^SmCl_3_ solution

The ^153^Sm was produced by neutron irradiation of 100 μg of enriched ^152^Sm_2_O_3_ according to reported procedures [11] at a thermal neutron flux of 5×10^13^ n.cm^–2^.s^−1^ for 5 days. Specific activity of the ^153^Sm was 27.75 GBq/mg. The irradiated target was dissolved in 200 μl of 1.0 mol/L HCl, to prepare ^153^SmCl_3_ and diluted to the appropriate volume with ultra pure water, to produce a stock solution. The mixture was filtered through a 0.22 μm biological filter and sent for use in the radiolableing step. Radionuclidic purity of the solution was tested for the presence of other radionuclides using beta spectroscopy and HPGe spectroscopy to detect various interfering beta and gamma emitting radionuclides. The radiochemical purity was also checked by Whatman No.2 chromatography paper, and developed in a mixture of 10 mmol/L DTPA solution as a mobile phase.

### Labeling maltolate with ^153^SmCl_3_

The labeling was developed in ethanolic media. Briefly, ^153^SmCl_3_ (111 MBq, 0.1 ml) was added to a borosilicate vial and dried by warming (50°C) under a nitrogen flow for about 15 minutes. Then, maltol (30mg, 0.25 mmol) dissolved in absolute ethanol (1 ml) was added to the dried residue and the mixture agitated and incubated at 60°C for 2 hours. The radiochemical purity of free samarium and Sm-MAL were determined by counting Whatman No.2 sheets as stationary phase using various mobile phases (A: ammonia:water:methanol (2:40:20), B: 1mM DTPA aqueous solution, C: %10 ammonium acetate:methanol system, 1:1). After obtaining the desired radiochemical purity, the ethanolic solution was concentrated by warming 40–50°C to 0.05 ml and then diluted to a 5% solution by adding 1 ml of normal saline.

### Stability testing of the radiolabeled compound in aqueous solution

Stability of [^153^Sm]-samarium maltolate in final preparation was determined by storing the final solution at 25°C for 24 h and performing frequent ITLC analysis using ammonia:water:methanol (2:40:20) mobile phase to determine radiochemical purity.

### Stability of [^153^Sm]-samarium maltolate in presence of human serum

Final [^153^Sm]-samarium maltolate solution (7.5 MBq, 50 μl) was incubated in the presence of freshly prepared human serum (300 μl) and kept at 37°C for 2 days. The complex stability was assessed by performing frequent ITLC analysis using ammonia:water:methanol (2:40:20) mobile phase to determine radiochemical purity.

### Determination of Partition coefficient

The partition coefficient of the [^153^Sm]-samarium maltolate was measured following 1 min of vigorous vortex mixing of 1 ml of 1-octanol and 1 ml of isotonic acetate-buffered saline (pH=7) with approximately 3.7 MBq of the radiolabeled complex at 37°C. Following further incubation for 5 min, the octanol and aqueous phases were sampled and counted in an automatic well counter. A 500 μl sample of the octanol phase from this partitioning was repartitioned two to three times with fresh buffer to ensure that traces of hydrophilic ^153^Sm impurities did not alter the calculated *P* values. The reported log *P* values are the average of the second and third extractions from three to four independent measurements, log *P* values represent the mean (standard deviation) of five measurements.

### Biodistribution of [^153^Sm]-samarium maltolate and ^153^SmCl_3_ in normal rats

To determine comparative biodistribution, [^153^Sm]-samarium maltolate and ^153^SmCl_3_ were administered to normal rats in separate groups (n=3). A volume (100–120 μl) of final [^153^Sm]-samarium maltolate solution (4800±185 KBq) radioactivity was injected intravenously to rats through their tail vein. The animals were sacrificed at the exact time intervals (2, 4, 24 hours and 48 h), and specific activity of different organs was calculated as percentage of injected dose per gram using HPGe detector.

## Conclusion

In this work, [^153^Sm]-samarium maltolate was prepared using Sm-153 chloride (radiochemical purity >99.9%; ITLC and specific activity). The stability of the labeled compound was determined in the final solution up to 24h as well as the partition coefficient. The partition coefficient for the labeled compound was calculated (logP. 1.869). Biodistribution studies of Sm-153 chloride and [^153^Sm]-samarium maltolate were carried out in wild-type rats comparing the critical organ uptakes. Comparative studies for Sm^3+^ cation and the labeled compound were conducted up to 48 h, demonstrating a more rapid wash out of activity for the labeled compound. The effective half life of about 2.3 h was calculated for the complex and the biological half life was 2.46 h. The radioactivity in case of the labeled compound is significantly removed from the blood and bone. The data suggests the detoxification property of maltol formulation for lanthanide overdoses.

## Figures and Tables

**Fig. 2.**
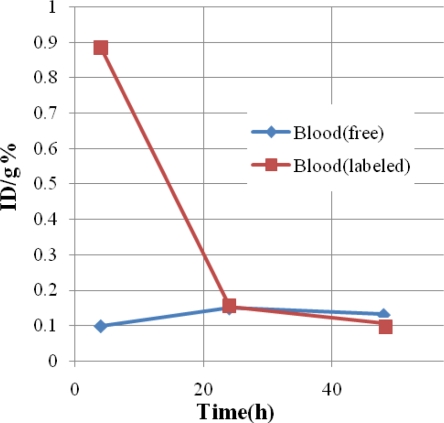
Comparative %ID/g in the blood for [^153^Sm]-samarium maltolate (labeled, red) and ^153^SmCl_3_ (free, blue) in wild-type rats

**Fig. 6.**
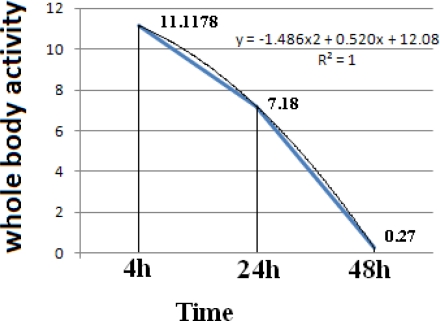
Whole-body activity content/time curve for [^153^Sm]-samarium maltolate in 4–48 time period in wild-type rats

**Tab. 1.** Chromatographic properties of [^153^Sm]-samarium chloride and [^153^Sm]-samarium maltolate

**Chromatographic systems**	**R_f_** **for [^153^Sm]-samarium chloride**	**R_f_** **for [^153^Sm]-samarium maltolate**
DTPA solution (pH. 5), Whatman no. 2	0.8	0.05
10% ammonium acetate:methanol, Si	0.01	0.3
ammonia: water: methanol (2:40:20), Whatman no. 2	0.05	0.8

**Tab. 2.** Purities for radioactive samples used in this study (n=5)

**Purity (%)**	**[^153^Sm]-samarium chloride**	**[^153^Sm]-samarium maltolate**
Radiochemical	98±0.2	>99
Chemical	>99	>99
Radionuclidic	>99.9	>99.9

**Tab. 3.** Percentage of injected dose per gram (ID/g %) of ^153^SmCl_3_ in wild-type rat tissues at 2, 4, 24 and 72 h post injection

**Organs**	**Injected dose per gram (ID/g %±SD) [^153^Sm]-samarium chloride**			
	**2h**	**4 h**	**24 h**	**48h**
blood	0.07±0.0676	0.0980±0.006	0.1493±0.0096	0.1302±0.09
heart	0.26±0.0068	0.2404±0.0361	0.1495±0.0114	0.0778±0.011
liver	4.23±0.21	4.1033±0.0686	3.1496±0.0888	3.1192±0.09
kidney	1.3±0.117	1.2594±0.0856	1.3806±0.0404	1.2674±0.06
skin	0.04±0.02	0.0656±0.0168	0.0503±0.0101	0.0525±0.01
muscle	0.02±0.01	0.0218±0.0057	0.0132±0.0026	0.0109±0.02
bone	1.16±0.015	1.1450±0.0740	1.1231±0.0581	0.8606±0.06
intestine	0.1±0.01	0.0980±0.0205	0.0789±0.0245	0.0750±0.03
lung	0.23±0.04	0.2157±0.0162	0.1553±0.0697	0.1006±0.05
spleen	0.65±0.05	0.6084±0.0200	0.4629±0.0788	0.2179±0.04
brain	0.004±0.001	0.0090±0.0011	0.0374±0.0023	0.0137±0.002
stomach	0.18±0.09	0.1978±0.0064	0.0669±0.0045	0.1620±0.003

**Tab. 4.** Percentage of injected dose per gram (ID/g %±SD) of [^153^Sm]-samarium maltolate in wild-type rat tissues at 2, 4, 24 and 72h post injection

**Organs**	**Injected dose per gram (ID/g %±SD) [^153^Sm]-samarium maltolate**			
	**2h**	**4h**	**24h**	**48h**
Blood	1.0969±0.5547	0.8861±0.0106	0.1543±0.0105	0.0607±0.0060
Heart	0.6494±0.0320	0.0694±0.0027	0.1351±0.0028	0.2426±0.0269
Lung	0.5423±0.0253	0.5717±0.0100	0.2018±0.0140	0.0980±0.0064
Stomach	0.0874±0.0013	0.1846±0.002	0.0754±0.0013	0.0175±0.0013
Colon	0.0335±0.0020	0.1152±0.005	0.0273±0.002	0.0249±0.0025
Intestine	0.1446±0.0050	0.1599±0.002	0.0517±0.0041	0.0042±0.0003
Liver	4.6164±0.1846	6.1051±0.10004	3.2293±0.0779	0.8590±0.0350
Spleen	1.0166±0.0033	1.3231±0.0085	1.5545±0.0744	0.1482±0.003
Kidney	0.8232±0.0139	0.7729±0.01405	0.7591±0.0060	0.5676±0.045
Muscle	0.0753±0.0023	0.0261±0.002	0.0842±0.0013	0.0446±0.0017
Sternum	0.1938±0.0102	0.2968±0.01074	0.1601±0.002	0.6909±0.0204
Thigh bone	0.4438±0.0255	0.5590±0.04013	0.7005±0.007	0.0896±0.0010
Skin	0.0390±0.0015	0.0473±0.01085	0.0491±0.0103	0.1494±0.0061
Brain	0±0.00	0±0.00	0±0.00	0±0.00

## References

[1] Bernstein LR, Tanner T, Godfrey C, Noll B (2000). Chemistry and Pharmacokinetics of Gallium Maltolate, a compound with high oral Gallium Bioavailability. Metal Based Drugs.

[2] Thompson KH, Barta CA, Orvig C (2006). Metal complexes of maltol and close analogues in medicinal inorganic chemistry. Chem Soc Rev.

[3] Chitambar CR, Purpi DP, Woodliff J, Yang M, Wereley JP (2007). Development of Gallium Compounds for Treatment of Lymphoma: Gallium Maltolate, a Novel Hydroxypyrone Gallium Compound, Induces Apoptosis and Circumvents Lymphoma Cell Resistance to Gallium Nitrate. J Pharm Exp Ther.

[4] Satoh E, Yasuda I, Yamada T, Suzuki Y, Ohyashiki T (2007). Involvement of NO Generation in Aluminum-Induced Cell Death. Biol Pharm Bull.

[5] DeLeon K, Balldin F, Watters C, Hamood A, Griswold J, Sreedharan S, Rumbaugh K (2009). Gallium Maltolate Treatment Eradicates Pseudomonas aeruginosa Infection in Thermally Injured Mice. Antimicr Agents Chemother.

[6] Liu ZD, Piyamongkol S, Liu DY, Khodr HH, Lu SL, Hider RC (2001). Synthesis of 2-amido-3-hydroxypyridin-4(1H)-ones: novel iron chelators with enhanced pFe3+ values. Bioorg Med Chem.

[7] Finnegan MM, Lutz TG, Nelson WO, Smith A, Orvig C (1987). Neutral water-soluble post-transition-metal chelate complexes of medical interest: aluminum and gallium tris(3-hydroxy-4-pyronates). Inorg Chem.

[8] Monga V, Patrick BO, Orvig C (2005). Group 13 and lanthanide complexes with mixed O,S anionic ligands derived from maltol. Inorg Chem.

[9] Firestone RB, Shirley VS, Baglin CM, Zipkin J (1996). Table of isotopes.

[10] Misra SN, Gagnani MA, Shukla RS (2004). Biological and clinical aspects of lanthanide coordination compounds. Bioinorg Chem Appl.

[11] Ferro-Flores G, De Mara Ramrez F, Tendilla JI, Pimentel-Gonzlez G, Murphy CA, Melndez-Alafort L, Ascencio JA, Croft BY (1999). Preparation and Pharmacokinetics of Samarium(III)-153-Labeled DTPA-bis-Biotin. Characterization and Theoretical Studies of the Samarium(III)-152 Conjugate. Bioconjug Chem.

